# The Clinico-Epidemiological Profile of Patients with Gonorrhoea and Challenges in the Management of Neisseria gonorrhoeae Infection in an STI clinic, Ternopil, Ukraine (2013-2018)

**DOI:** 10.25122/jml-2019-0170

**Published:** 2020

**Authors:** Iryna Boiko, Viorika Akimova, Lyudmyla Mazur, Iryna Savchenko, Ihor Kohut, Inna Krynytska

**Affiliations:** 1. Department of Functional and Laboratory Diagnostics, I. Horbachevsky Ternopil National Medical University, Ternopil, Ukraine; 2. Department of Clinical Laboratory Diagnostic, Danylo Halytsky Lviv National Medical University, Lviv, Ukraine; 3. Department of Clinical Immunology, Allergology and General Patients’ Care, I. Horbachevsky Ternopil National Medical University, Ternopil, Ukraine;; 4. First Department of Internal Medicine, I. Horbachevsky Ternopil National Medical University, Ternopil, Ukraine;; 5. Department of Infectious Diseases with Epidemiology, Skin and Sexually Transmitted Diseases, I. Horbachevsky Ternopil National Medical University, Ternopil, Ukraine.

**Keywords:** *Neisseria gonorrhoeae*, gonorrhea, sexually transmitted infections, epidemiology, management

## Abstract

Gonorrhea is the second most common sexually transmitted infection spreading worldwide and a serious public health problem. However, further data are required to improve the management of gonorrhea.

Our aim was to review the features of gonococcal infection and characterize the challenges of its management.

A retrospective descriptive study of the medical records of 136 adult patients with gonorrhea that visited Ternopil Regional Sexually Transmitted Infections Clinic (Ukraine) in 2013-2018 was performed.

The male-to-female ratio was 6.6:1. Homosexually-acquired gonorrhoea was 3.7%. Also, most patients acquired gonorrhea in Ukraine (98.4%). The mean infectious period lasted 2-16 days, including the incubation period of 1-9 days and the period from the onset of symptoms to the first visit of the clinic of 1-7 days. The probability of *N. gonorrhoeae* transmission within the frame of the epidemiologic sexual chain was 1:2.4. Concurrent *T. vaginalis* (39.7%) and *C. trachomatis* (2.2%) were detected. HIV and syphilis screening rates were 1.6% and 0.7%, respectively. The examining rate of sexual partners was 11%, testing extragenital specimens - 0.7%, screening coverage for HIV - 46.3%, compliance with follow-up visits - 41.9%. Part of patients (16.2%) received monotherapy with clarithromycin, doxycycline, benzylpenicillin, azithromycin, or ofloxacin.

The management of *N. gonorrhoeae* infections was compromised by a low rate of examining sexual partners, females and testing extragenital specimens, screening for HIV, compliance to follow-up visits, access to nucleic acid amplification tests, and receiving questionable or even obsolete antimicrobial treatment. Therefore, more accurate and comprehensive management of gonorrhea is urgently needed in Ukraine.

## Introduction

Gonorrhea is the second most common sexually transmitted infection (STI) spreading worldwide [1-2]. It is a serious public health problem causing severe secondary sequelae, including pelvic inflammatory disease, first-trimester miscarriages, ectopic pregnancy, and infertility. *N. gonorrhoeae* infection may facilitate HIV acquisition and transmission [3-5]. Having been treated successfully before, nowadays, gonorrhea is considered to be one of the challenging STI due to the extraordinary ability of *N. gonorrhea* to develop resistance to almost all antimicrobials [6].

In 2018, the annual data of the Centre of Medical Statistics of the Ministry of Health reported that the incidence of gonorrhea has decreased to 9.7/100.000 and 9.2/100.000 population of Ukraine and Ternopil, respectively, which was 2.8 and 2.5 times lower than reported in 2008. Many cases of gonorrhea are not registered officially, and the real prevalence remains unknown in Ukraine [7]. Conventional, microscopy, and culture, which are less sensitive compared with molecular tests, are used preferably to detect gonorrhea in Ukraine [8].

Studying the socio-demographic, epidemiological, clinical and laboratory diagnostics data of patients with gonorrhea could highlight the main issues in the management of *N. gonorrhea* infection at the Ternopil regional STI clinic in Ukraine in 2013-2018.

## Material and Methods

A retrospective single-center descriptive study was performed at a tertiary dermatology and venerology care center, Ternopil Regional STI Clinic, Ukraine, from July 2013 to August 2018. One-hundred-thirty-six adult patients with gonorrhea were enrolled after giving their written informed consent. All personal information used in the study was confidential, and patient's identification information was not disclosed. Patients and their sexual partners were managed according to the national guidelines. Diagnosis of gonorrhea, urogenital chlamydiosis, and trichomoniasis was made as it was previously described in the collaborative project [8, 9]. Urogenital smears were stained by methylene blue and Gram for microscopic detection of *Gardnerella vaginalis* and *Candida spp*. *Herpes simplex type* *2* was recognized by enzyme-linked immunosorbent assay for detection of specific IgG (Vitrotest HSV2-IgG reagent kit, Ramintek Ltd., Ukraine) and by microscopy using Romanovsky-Giemsa-stained smears. The syphilis status was determined by the reaction of microprecipitation using cardiolipin antigen (Public Joint-Stock Company “Pharmstandard-Biolik”, Ukraine) and Wasserman reaction using cardiolipin antigen for the complement fixation reaction, dried complement and diagnostic hemolytic serum for the complement fixation reaction (Public Joint-Stock Company “Pharmstandard-Biolik”, Ukraine).

The HIV-status was determined according to the European guidelines by using point-of-care tests - SD Bioline HIV ½ -3.0 (Standard Diagnostics, Inc., Korea) and Alere DetermineTM HIV-1/2 SET (Alere Medical Co., Ltd, Japan) [10]. Anogenital warts were diagnosed according to the 2012 European guidelines [11]. Molluscum contagiosum was diagnosed according to the guideline of the British Association for Sexual Health and HIV [12]. The medical records of all patients were reviewed to collect relevant epidemiological and clinical data.

### Analysis

The analyzed data referred to age, gender, residency, occupation, marital status, reported national/regional incidence of infection, history of STI, the onset of sexual activity, sexual behavior, time after the last sexual contact, number of sexual partners during the previous three months, sexual contacts examination, incubation period, number of days with symptoms before the first visit to the STI clinic, symptoms, clinical diagnosis, concomitant STI, the use of laboratory methods, syphilis and HIV status, microscopy results, treatment prescribed for gonorrhea at the clinic, and treatment outcomes. The 95% confidence interval (95% CI) was calculated using the exact binomial distribution method. P-value was displayed with a threshold at 0.05. All statistical analyses were conducted using the MedCalc Statistical Software version 18.11.3 (MedCalc Software bvba, Ostend, Belgium).

### Ethical approval

The Bioethics Commission of I. Horbachevsky Ternopil National Medical University approved the study (Excerpts from Minutes No. 29, dated 20.05.2015).

## Results

All 136 patients with gonorrhea included 86.8 % males and 13.2 % females. The male-to-female ratio was 6.6:1. The mean age of patients was 29.4 years (range 16 - 80). The majority of patients were residents of Ukraine (94.9%), urban citizens (71%), and single (75.6%). Detailed socio-demographic characteristics are presented in Table 1.

**Table 1:** Population characteristics of adult patients with gonorrhoea in Ternopil region of Ukraine, 2013-2018.

Population characteristics	All				Male			Female		p-value
N	%	[95%CI]	N	%	[95%CI]	N	%	[95%CI]	
Age, years	n=134									
< 25 years	44	32.8	[24.9-41.4]	37	31.6	[23.3-40.8]	7	41.2	[18.5-67.1]	0.4326
25-34years	60	44.8	[36.2-53.6]	51	43.6	[34.4-53]	9	52.9	[29.5-78.6]	0.4729
> 34 years	30	22.4	[15.7-30.4]	29	24.8	[17.3-33.6]	1	5.9	[0.2-28.7]	0.0819
**Country of origin**	n=136				n=118			n=18		
Ukraine	129	94.9	[59.9-76.1]	111	94.1	[88.2-97.6]	18	100	[81.5-100]	0.2918
Foreigner	7	5.1	[2.1-10.3]	7	5.9	[2.4-11.8]	0	0	[0-8.5]	0.2918
p-value	< 0.0001			< 0.0001			NA			
**Place of residence**	n=131				n=116			n=17		
Urban	93	71.0	[62.4-78.6]	81	69.8	[60.6-78]	14	82.4	[56.6-96.2]	0.2847
Rural	38	29.0	[21.4-37.6]	35	30.1	[21.9-39.3]	3	17.6	[3.8-43.4]	0.2881
p-value	< 0.0001			< 0.0001			0.0002			
Occupation	n=85				n=72			n=13		
Students	18	21.2	[13.1-31.4]	13	18.1	[10-28.9]	5	38.5	[13.9-68.5]	0.0998
Employed	12	14.1	[7.5-23.3]	10	13.9	[6.9-24.1]	2	15.4	[1.9-5.5]	0.8870
Unemployed	55	64.7	[53.6-74.8]	49	68.1	[56.1-78.6]	6	46.1	[19.2-74.8]	0.1288
**Marital status**	n=131				n=113			n=18		
Married	32	24.4	[17.3-32.7]	27	23.9	[16.4-32.8]	5	27.8	[9.7-3.5]	0.7216
Single	99	75.6	[67.3-82.7]	86	63.7	[54.1-72.5]	13	72.2	[46.5-90.3]	0.4846
**p-value**	**<0.0001**				**< 0.0001**			**0.0086**		

Note: CI, confidence interval; NA, not applicable.

Most of the patients were heterosexuals (96.3%), and only 3.7% of patients reported themselves as men-who-have-sex-with-men (MSM). The majority of patients had occasional partners (62.1%). Most patients (75%) had 2 - 4 sexual partners during the last three months.

The total sexual-chain comprised 400 persons. Those included 136 studied patients with gonorrhea and 264 sexual partners in the previous three months period. Less than half of persons from the sexual-chain group were examined (41.3%), including 136 patients with gonorrhea (34%) and 29 sexual partners (7.3%). The majority of sexual partners (89%) were not examined. Gonorrhea was detected in 41.4% (12/29) of the examined sexual partners. The probability of *N. gonorrhea* transmission within the frame of the epidemiologic sexual chain was high (1:2.4).

Most of the patients (98.4%) reported Ukraine as being the country of gonorrhea acquisition, mostly specifying the Ternopil region in 92.9% of cases. Females reported the Ternopil region as an area of acquiring gonorrhea significantly more frequently than males (p=0.031).

Sixteen patients (13.9%) reported a history of previous STI, including gonorrhea (5.1%), chlamydiosis (3.7%), trichomoniasis (6.6%), and syphilis (1.5%). The detailed epidemiologic characteristics of patients with gonorrhea are presented in Table 2.

**Table 2:** Epidemiological data of adult patients with gonorrhoea in the Ternopil region, Ukraine, 2013-2018.

Patient's characteristics	All			Male			Female			p-value
N	%	[95%CI]	N	%	[95%CI]	N	%	[95%CI]	
Relationship to the source of last sexual contact	n=132			n=115			n=17			
Occasional partner	82	62.1	[53.3-70.4]	101	87.8	[80.4-93.2]	8	47.1	[23-72.2]	< 0.0001
Constant partner	50	37.9	[29.6-46.8]	14	12.2	[6.8-19.6]	9	52.9	[27.8-77]	< 0.0001
p-value	0.0071			< 0.0001			0.7390			
**Number of sexual partners during the last 3 months**	n=136			n=118			n=18			
1	34	25	[18-33.1]	25	21.2	[14.2-29.7]	9	50	[26-74]	0.0088
2	80	58.8	[50-67.2]	76	64.4	[55.1-73]	4	22.2	[6.4-47.6]	0.0007
3	18	13.2	[8-20.1]	15	12.7	[7.3-20.1]	3	16.7	[3.6-41.5]	0.6420
4	4	3	[0.8-7.4]	2	1.7	[0.2-6]	2	11.1	[1.4-34.7]	0.0286
Sexual contact examination	n=264			n=230			n=34			
Examined	29	11	[7.5-15.4]	16	7	[4.1-11.1]	13	38.2	[22.1-56.4]	< 0.0001
Not examined	235	89	[84.6-92.5]	214	93	[88.9-95.9]	21	61.8	[43.6-77.9]	< 0.0001
p-value	< 0.0001			< 0.0001			0.0534			
Reported country of infection	n=129			n=111			n=18			
Ukraine	127	98.4	[94.6-99.8]	109	98.2	[93.7-99.8]	18	100	[81.5-100]	0.5677
Abroad	2	1.6	[0.2-5.4]	2	1.8	[0.2-6.4]	0	0	[0-8.5]	0.5677
p-value	< 0.0001			< 0.0001			NA			
Reported region of infection	n=127			n=109			n=18			
Ternopil	118	92.9	[87-96.7]	100	78.7	[69.8-86]	18	100	[81.5-100]	0.0310
Other regions of Ukraine (Kyiv, Rivne, Lviv, Sumy, Lutsk)	9	7.1	[3.3-13]	9	21.3	[14-30.2]	0	0	[0-8.5]	0.0310
p-value	< 0.0001			< 0.0001			NA			
History of STI	n=115			n=97			n=18			
Any STI previously	99	86.1	[78.4-91.8]	83	85.6	[77-91.9]	16	88.9	[65.3-98.6]	0.7112
Reported STI history	16	13.9	[8.2-21.6]	14	14.4	[8.1-23]	2	11.1	[1.4-34.7]	0.7112
p-value	< 0.0001			< 0.0001			< 0.0001			

Note: CI, confidence interval; MSM, men who have sex with men; STI, sexually transmitted infections; NG, *Neisseria gonorrhoeae*; CT, *Chlamydia trachomatis*; TV, *Trichomonas vaginalis*; NA, not applicable.

The mean incubation period of gonorrhea was 4.6 days (ranging from 1 to 9). The patients visited the STI clinic in 4.4 days (range: 1-7) after the first symptoms of gonorrhea. A total mean of the infectious period from the infectious sexual intercourse to the first clinic visit (start of treatment) was 9 days (range: 2-16). Males were symptomatic in 94.1% and females in 61.1% of cases. The most prevalent symptoms were urethral discharge (78.8%) in males and vaginal discharge (38.9%) in females. Non-specific symptoms were reported by 14.4% male and 22.2% female patients. Detailed clinical characteristics are shown in Table 3.

**Table 3:** Clinical characteristics of adult patients with gonorrhoea in the Ternopil region, Ukraine, 2013-2018

Patient's characteristics	All		
N	%	[95%CI]
Male symptoms	n=118		
Urethral discharge	93	78.8	[70.3-85.8]
Urethral burning sensation with urination	83	70.3	[61.2-78.4]
Other (rash on genitalia: n=8; unpleasant smell: n=4; swelling and redness of the glans penis: n=2; lower abdominal pain: n=1; frequent urination: n=1; itching of the penis: n=1)	17	14.4	[8.6-22.1]
Male clinical diagnosis	n=118		
Urethritis	112	95	[89.4-98.2]
Balanitis	7	5.9	[2.4-11.8]
Balanoposthitis	4	3.4	[0.9-8.5]
Proctitis	1	0.8	[0.02-4.6]
Female symptoms	n=18		
Vaginal discharges	7	38.9	[17.3-64.3]
Other (vaginal itching: n=1; rash on genitalia: n=1; discomfort during urination: n=1; unpleasant smell: n=1)	4	22.2	[6.4-47.6]
Female clinical diagnosis	n=18		
Endocervicitis	13	72.2	[46.5-90.3]
Colpitis	6	33.3	[13.3-59]
Cervical erosion	3	16.7	[3.6-41.5]

Note: CI, confidence interval

Concurrent STIs were detected in 45.6% of patients, being represented by *T. vaginalis* in 39.7%, *C. trachomatis* in 2.2%, anogenital warts in 2.2%, *Gardnerella vaginalis* in 2.2%, *Herpes simplex type* *2* in 1.5%, molluscum contagiousum in 0.7%, and *Candida spp*. in 1.6% of cases. HIV testing was performed in 46.3% of patients, and a positive rate was found in 1.6% of cases. The rest of the patients (53.7%) refused to be tested for HIV. All patients were screened for syphilis, and 0.7% positive results were received.

*N. gonorrhoeae* isolates were obtained from the male urethra in 86% specimens, from the cervix in 13.2%, from the rectum of MSM in 0.7% of cases. No specimen was collected from the pharynx.

Ceftriaxone-based therapy was used for 83.8% of patients. Dual therapy was administered the most frequently, based on ceftriaxone combined with other antimicrobials (67%). Monotherapy by ceftriaxone 1 g was used in 16.8% of cases. Nevertheless, 16.2% of patients were not treated with ceftriaxone, receiving monotherapy instead with clarithromycin, doxycycline, benzylpenicillin, azithromycin, or ofloxacin. Recovery was detected in all patients who had follow-up visits (58.1%), but 41.9% of patients ignored their follow-up testing.

## Discussion

Gonorrhea is spreading worldwide, and its prevalence is rising, especially in well-developed countries, as proved by highly sensitive and specific modern laboratory methods for screening and diagnosis [1-2]. However, the incidence of gonorrhea has dropped down in Ukraine during the last decade [7]. Nowadays, the management of gonorrhea remains quite challenging [13]. Population, epidemiological, clinical, and diagnostic data were analyzed to reveal the main existing challenges of management of *N. gonorrhea* infection in Ukraine.

In our study, males were examined disproportionally more often than females, and the male to female ratio was 6.6:1. Dave and Tshokey et al. presented almost the same rate of gonorrhea in females [14-15], while Giomi B. et al. reported a higher level in males [16]. Our data suggest that a majority of potentially infected females probably have been missed.

Homosexually-acquired gonorrhea was only 3.7% of the total. Other authors reported a higher level of MSM (25.9 - 52%) and bisexuals (1%) in STI clinics [14,16]. Our study supposes that patients in Ukraine have a stigma to inform about their sexual behavior.

According to our study, gonorrhea had been a local infection during 2013-2018, because most patients were residents and reported Ukraine and the Ternopil region as the geographical area where they acquired the infection. Giomi B. et al. reported that the same data was received in STI clinics in Italy in 2001 [16]. A significant level of antimicrobial resistance of *N. gonorrhoeae* has been reported in Europe as well as over the world [17-20]. Therefore, exporting of gonococcal infections in the future can not be excluded, including antimicrobial-resistant isolates of *N. gonorrhea*.

According to the European gonorrhea guidelines, all sexual contacts of patients with gonorrhea should be traced within at least the preceding two months of symptoms onset, and three months after the diagnosis [21]. Our study showed that only 11% of the sexual partners passed the medical examination, and gonorrhea was detected in 41.4% of partners, which indicates a high probability of *N. gonorrhea* transmission (1:2.4) within the frames of epidemiologic sexual chains. Worryingly, 89% of sexual contacts were not examined, guessing that almost a half could be infected with *N. gonorrhoeae*.

A mean infectious period from the infectious sexual intercourse to the first visit at the clinic before the patients were diagnosed and started treatment was comparatively long (2-16 days). This fact may facilitate a transferring of gonococcal infection. Worryingly, this also could provoke a self-treatment because antimicrobials are over-the-counter drugs in Ukraine and available even through electronic marketing.

Asymptomatic gonorrhea was found in 5.9% males, and 38.9% of females and non-specific symptoms were expressed in 14.4% and 22.2% of cases, respectively. Asymptomatic patients could remain the hidden source of *N. gonorrhea* transmission, mainly if low sensitivity laboratory tests are used for screening and detection, i.e., microscopy and culture [8, 21].

Extragenital samples were taken extremely rare, as only one rectal sample was registered in this study. Information on oral-genital intercourses was not gathered, and pharyngeal samples were not collected in this study. This indicates a present stigma in patients to inform about the type of sexual intercourse and sexual behavior. Therefore, clinicians should collect more detailed sexual history and take extragenital samples, especially pharyngeal, that are considered to be hidden sites of *N. gonorrhea*, where antimicrobial resistance may develop [21].

The most prevalent concurrent STI infection among patients with gonorrhea is *C. trachomatis* [2]. A comparatively low rate of chlamydial infection (2.2%) was registered by using conventional laboratory tests (microscopy by Romanovsky-Giemsa and serology by ELISA), indicating a crucial underdiagnosis of *C. trachomatis* [8]. The highly effective molecular test is urgently needed to be implemented into the routine STI diagnostic in Ukraine, as well it is used in most countries worldwide [23-25].

A significant part of studied patients had a high level of concomitant *T. vaginalis* (39.7%) detected by microscopy. Previously, we described suboptimal performance characteristics for methylene blue and Gram-stained microscopy [8]. The present study confirmed that highly specific, sensitive, and quality assured molecular tests are needed for routine STI diagnostic in Ukraine.

Worryingly, 53.7% of observed patients were not tested for HIV, as it was the same reported in other studies [26]. Li et al. suggest that a high level of HIV infection is present in patients with STI and particularly with gonorrhea [27]. Our study highlighted that improving HIV counseling is needed among patients with gonorrhea in Ukraine.

Nevertheless, the concurrent syphilis was detected in 0.7% of patients with gonorrhea; the real level of *T. pallidum* infection is supposed to be underestimated because of using non-treponemal tests during screening [28, 29]. Accordingly, a highly sensitive and quality assured syphilis serology tests are imperative in Ukraine.

A significant part of patients (41.9%) who missed their follow-up visits could form a reservoir of persistent infection and be a source of further transmission of *N. gonorrhea*.

Some patients (16.2%) received questionable treatment using monotherapy with clarithromycin, doxycycline, benzylpenicillin, azithromycin, or ofloxacin [21]. Ukrainian gonorrhea treatment guidelines were not updated since 2009. It is essential to make further improvement of treatment based on the European gonorrhea guidelines or adapted national evidence-based guidelines [9, 21, 23, 24].

An issue of social stigma associated with gonorrhea was raised previously [1, 15]. Our study supposed that gonococcal infection is stigmatized in Ukraine as well because of low levels of (a) reporting homosexual behavior, (b) willing to be screened for HIV, (c) compliance to have follow-up visits, and sporadically collection of extragenital samples by clinicians.

## Conclusions

The key challenges of the management of *N. gonorrhoeae* infection in Ukraine are the low level of examined females, asymptomatic infection in patients and sexual partners; disregarded testing of extragenital specimens, using of conventional laboratory tests (microscopy and culture) for screening and diagnostics of gonorrhea; deficient access to nucleic acid amplification tests in the public leading specialised STI clinics in Ukraine, low level of screening for HIV; low compliance of the follow-up visits; receiving questionable or even obsolete antimicrobial treatment. It is also supposed by our data that gonococcal infection is stigmatised. Therefore, more accurate and comprehensive management of gonorrhoea is urgently needed in Ukraine.

## Conflict of Interest

The authors confirm that there are no conflicts of interest.

## References

[1] Unemo M, Seifert HS, Hook EW, Hawkes S, Ndowa F, Dillon JR (2019). Gonorrhoea. Nat Rev Dis Primers.

[2] Rowley J, Vander Hoorn S, Korenromp E, Low N, Unemo M, Abu-Raddad LJ, Chico RM, Smolak A, Newman L, Gottlieb S, Thwin SS, Brouteta N, Taylora MM (2019). Chlamydia, gonorrhoea, trichomoniasis and syphilis: global prevalence and incidence estimates, 2016. Bull World Health Organ.

[3] Sánchez-Busó L, Golparian D, Corander J, Grad YH, Ohnishi M, Flemming R, Parkhill J, Bentley SD, Unemo M, Harris SR (2019). The impact of antimicrobials on gonococcal evolution. Nat Microbiol.

[4] Tsevat DG, Wiesenfeld HC, Parks C, Peipert JF (2017). Sexually transmitted diseases and infertility. Am J Obstet Gynecol.

[5] Koval HD, Chopyak VV, Kamyshnyi OM, Kurpisz MK (2018). Transcription regulatory factor expression in T-helper cell differentiation pathway in eutopic endometrial tissue samples of women with endometriosis associated with infertility. Cent Eur J Immunol.

[6] Unemo M, Golparian D, Eyre DW (2019). Antimicrobial Resistance in Neisseria gonorrhoeae and Treatment of Gonorrhea. Methods Mol Biol.

[7] Mavrov GI, Bondarenko GM (2002). The evolution of sexually transmitted infections in the Ukraine. Sex Transm Infect.

[8] Boiko I, Golparian D, Krynytska I, Unemo M (2019). High prevalence of Chlamydia trachomatis, *Neisseria gonorrhoeae* and particularly Trichomonas vaginalis diagnosed using US FDA-approved Aptima molecular tests and evaluation of conventional routine diagnostic tests in Ternopil, Ukraine. APMIS.

[9] Boiko I, Golparian D, Krynytska I, Bezkorovaina H, Frankenberg A, Onuchyna M, Jacobsson S, Unemo M (2019). Antimicrobial susceptibility of Neisseria gonorrhoeae isolates and treatment of gonorrhoea patients in Ternopil and Dnipropetrovsk regions of Ukraine, 2013-2018. APMIS.

[10] Gökengin D, Geretti AM, Begovac J, Palfreeman A, Stevanovic M, Tarasenko O, Radkliffe K (2014). 2014 European Guideline on HIV testing. Int J STD AIDS.

[11] Lacey CJ, Woodhall SC, Wikstrom A, Ross J (2013). 2012 European guideline for the management of anogenital warts. J Eur Acad Dermatol Venereol.

[12] Fernando I, Pritchard J, Edwards SK, Grover D (2015). UK national guideline for the management of Genital Molluscum in adults, 2014 Clinical Effectiveness Group, British Association for Sexual Health and HIV. Int J STD AIDS.

[13] Unemo M, Bradshaw CS, Hocking JS, de Vries HJC, Francis SC, Mabey D, Marrazzo JM, Sonder GJ, Schwebke JR, Hoornenborg E, Peeling RW, Philip SS, Low N, Fairley CK (2017). Sexually transmitted infections: challenges ahead. Lancet Infect Dis.

[14] Dave J, Paul J, Pasvol TJ, Williams A, Warburton F, Cole K, Miari VF, Stabler R, Eyre DW (2019). Ethnically diverse urban transmission networks of *Neisseria gonorrhoeae* without evidence of HIV serosorting. Sex Transm Infect.

[15] Tshokey T, Tshering T, Pradhan AR, Adhikari D, Sharma R, Gurung K, Dorji T, Wangmo S, Dorji U, Wangdi K (2018). Antibiotic resistance in Neisseria gonorrhoea and treatment outcomes of gonococcal urethritis suspected patients in two large hospitals in Bhutan, 2015. PLoS One.

[16] Giomi B, Silvestri C, Bravi S, Foretic M, Zuccati G, Martini P, Bilenchi R, Vichi F, Voller F, Cipriani F (2015). Epidemiological and clinical characteristics of patients attending STI clinics in Tuscany, Italy: a multicenter report on new infections in 2011. G Ital Dermatol Venereol.

[17] Mlynarczyk-Bonikowska B, Malejczyk M, Majewski S, Unemo M (2018). Antibiotic resistance and NG-MAST sequence types of *Neisseria gonorrhoeae* isolates in Poland compared to the world. Postepy Dermatol Alergol.

[18] Buder S, Dudareva S, Jansen K, Loenenbach A, Nikisins S, Sailer A, Guhl E, Kohl PK, Bremer V, GORENET study group (2018). Antimicrobial resistance of *Neisseria gonorrhoeae* in Germany: low levels of cephalosporin resistance, but high azithromycin resistance. BMC Infect Dis.

[19] Jacobsson S, Golparian D, Cole M, Spiteri G, Martin I, Bergheim T, Borrego MJ, Crowley B, Crucitti T, Van Dam AP, Hoffmann S, Jeverica S, Kohl P, Mlynarczyk-Bonikowska B, Pakarna G, Stary A, Stefanelli P, Pavlik P, Tzelepi E, Abad R, Harris SR, Unemo M (2016). WGS analysis and molecular resistance mechanisms of azithromycin-resistant (MIC >2 mg/L) *Neisseria gonorrhoeae* isolates in Europe from 2009 to 2014. J Antimicrob Chemother.

[20] Cobo F, Cabezas-Fernández MT, Cabeza-Barrera MI (2016). Antimicrobial susceptibility and typing of *Neisseria gonorrhoeae* strains from Southern Spain, 2012-2014. Enferm Infecc Microbiol Clin.

[21] Bignell C, Unemo M (2013). 2012 European guideline on the diagnosis and treatment of gonorrhoea in adults. Int J STD AIDS.

[22] Kohl PK (2006). Gonorrhoe. Urologe A.

[23] Clarke E, Patel C, Patel R, Unemo M (2020). The 2018–19 International Union against Sexually Transmitted Infections European Collaborative Clinical Group report on the diagnosis and treatment of gonorrhoea in Europe. Int J STD AIDS.

[24] Unemo M, Clarke E, Boiko I, Patel C, Patel R (2020). ECCG Core Group. Adherence to the 2012 European gonorrhoea guideline in the WHO European Region according to the 2018–19 International Union against Sexually Transmitted Infections European Collaborative Clinical Group gonorrhoea survey. Int J STD AIDS.

[25] Jacobsson S, Boiko I, Golparian D, Blondeel K, Kiarie J, Toskin I, Peeling RW, Unemo M (2018). WHO laboratory validation of Xpert® CT/NG and Xpert® TV on the GeneXpert system verifies high performances. APMIS.

[26] Ito S, Hanaoka N, Shimuta K, Seike K, Tsuchiya T, Yasuda M, Yokoi S, Nakano M, Ohnishi M, Deguchi T (2016). Male non-gonococcal urethritis: From microbiological etiologies to demographic and clinical features. Int J Urol.

[27] Li R, Zhao G, Li J, McGoogan JM, Zhou C, Zhao Y, Liang Z, Zhang H, Zuo Y, Lan L, Wu Z (2018). HIV screening among patients seeking care at Xuanwu Hospital: A cross-sectional study in Beijing, China, 2011-2016. PLoS One.

[28] Janier M, Hegyi V, Dupin N, Unemo M, Tiplica GS, Potočnik M (2014). 2014 European guideline on the management of syphilis. J Eur Acad Dermatol Venereol.

[29] Unemo M, Ison C (2013). Gonorrhoea Laboratory diagnosis of sexually transmitted infections, including human immunodeficiency virus..

